# Predicting the mechanism and rate of H-NS binding to AT-rich DNA

**DOI:** 10.1371/journal.pcbi.1006845

**Published:** 2019-03-07

**Authors:** Enrico Riccardi, Eva C. van Mastbergen, William Wiley Navarre, Jocelyne Vreede

**Affiliations:** 1 Department of Chemistry, NTNU, Trondheim, Norway; 2 Van’t Hoff Institute for Molecular Sciences, University of Amsterdam, Amsterdam, The Netherlands; 3 Department of Molecular Genetics, University of Toronto, Toronto, Ontario, Canada; University of Virginia, UNITED STATES

## Abstract

Bacteria contain several nucleoid-associated proteins that organize their genomic DNA into the nucleoid by bending, wrapping or bridging DNA. The Histone-like Nucleoid Structuring protein H-NS found in many Gram-negative bacteria is a DNA bridging protein and can structure DNA by binding to two separate DNA duplexes or to adjacent sites on the same duplex, depending on external conditions. Several nucleotide sequences have been identified to which H-NS binds with high affinity, indicating H-NS prefers AT-rich DNA. To date, highly detailed structural information of the H-NS DNA complex remains elusive. Molecular simulation can complement experiments by modelling structures and their time evolution in atomistic detail. In this paper we report an exploration of the different binding modes of H-NS to a high affinity nucleotide sequence and an estimate of the associated rate constant. By means of molecular dynamics simulations, we identified three types of binding for H-NS to AT-rich DNA. To further sample the transitions between these binding modes, we performed Replica Exchange Transition Interface Sampling, providing predictions of the mechanism and rate constant of H-NS binding to DNA. H-NS interacts with the DNA through a conserved QGR motif, aided by a conserved arginine at position 93. The QGR motif interacts first with phosphate groups, followed by the formation of hydrogen bonds between acceptors in the DNA minor groove and the sidechains of either Q112 or R114. After R114 inserts into the minor groove, the rest of the QGR motif follows. Full insertion of the QGR motif in the minor groove is stable over several tens of nanoseconds, and involves hydrogen bonds between the bases and both backbone and sidechains of the QGR motif. The rate constant for the process of H-NS binding to AT-rich DNA resulting in full insertion of the QGR motif is in the order of 10^6^ M^−1^s^−1^, which is rate limiting compared to the non-specific association of H-NS to the DNA backbone at a rate of 10^8^ M^−1^s^−1^.

## Author summary

The Histone-like Nucleoid Structuring protein (H-NS) occurs in enterobacteria, such as *Salmonella typhimurium* and *Escherichia coli*, and structures DNA by forming filaments along DNA duplexes. Several nucleotide sequences have been identified to which H-NS binds with high affinity. Yet, obtaining highly detailed structural information of the H-NS DNA complex has proven to be a major challenge, which has not been yet resolved. By employing molecular dynamics simulations we were able to provide high resolution insights into the mechanism of DNA binding by H-NS. We identified various ways in which H-NS can bind to DNA. In all binding events, a conserved region in the protein initiates the association of H-NS to DNA. Our results show that H-NS binds in the minor groove of AT-rich DNA via a series of intermediate steps. Using advanced molecular simulation methods we predicted that the process of H-NS binding to the DNA backbone to full insertion into the minor groove occurs in the order of a million times per second, which is slower than the non-specific association of H-NS to the DNA backbone.

## Introduction

Bacterial chromosomal DNA is organized within the nucleoid, which is distinctly different from the cytoplasm. The organization of the nucleoid in bacteria involves a group of DNA binding proteins known as architectural proteins. The Histone-like Nucleoid Structuring protein (H-NS) is a architectural protein occurring in Gram-negative enterobacteria and plays a key role in the genome organization. H-NS can structure DNA by binding to two separate DNA duplexes or to adjacent sites on the same duplex, depending on external conditions [1–6]. In addition, H-NS is a global regulator of transcription as it binds to promoter regions [7–9]. As H-NS has a preference to bind to foreign genetic material, it functions as a xenogeneic silencer. Activation of foreign H-NS-silenced genes occurring in response to lethal environmental conditions links H-NS to bacterial stress resistance and virulence [10–13].

H-NS is a relatively small protein composed of 137 amino acid residues that comprises two domains: the oligomerization domain and the DNA binding domain. The first 83 residues represent the oligomerization domain and is composed of four helices [14]. This domain contains two sites for homodimerization and can multimerize into higher order structures [15]. At low concentrations, H-NS primarily exists as a dimer [1]. Residues 89-137 make up the DNA-binding domain, which consists of an antiparallel *β*-sheet, an *α* helix and a 3_10_ helix [15, 16]. NMR experiments on the full-length H-NS protein indicate that the oligomerization domain and DNA binding domain function independently, suggesting that a flexible linker connects the two [17]. This region, residues 65-93, contains the most divergent amino acid sequence of H-NS-related proteins and is composed of amino acids that typically occur in linkers [17, 18]. However, removing positively charged residues from the linker abrogates DNA binding by H-NS [19].

The loop (residues 112-114) between one *β* strand and the *α* helix of the DNA-binding domain contains a conserved three amino acid sequence: QGR [4, 17]. NMR experiments indicate that this motif interacts with the minor groove of DNA [16] in a manner similar to other H-NS related proteins, such as Ler and Lsr2 [16, 20, 21]. Gel electrophoresis experiments revealed that H-NS binds to curved DNA [22, 23]. Several experiments showed that H-NS prefers to bind to conserved nucleotide sequences that are rich in AT and tend to be curved [24–28]. Recent studies using either protein binding microarrays or chromatin immunoprecipitation reveal that H-NS has a high affinity for AT-rich sequences with short A-tracts interrupted by TpA steps [3, 10, 24, 28]. This is in agreement with previously reported high-affinity H-NS binding sites [25]. Varying the relative location of high affinity H-NS binding sites on plasmids with respect to each other results in different topologies of the resulting plasmid and H-NS complexes [29].

To date, highly detailed structural information on the H-NS-DNA complex is still lacking. Molecular simulation can complement experiments by providing information in atomistic detail. A coarse grained approach highlighted the relevance of protein flexibility in forming DNA bridges, yet lacks full atomistic insights [30, 31], that are necessary in order to consider eventual structural modifications of complexes [32, 33]. All-atom molecular dynamics (MD) simulations follow a molecular system in time, and can provide the required resolution in both space and time to characterize the binding of H-NS to DNA. Recently, an MD exploration of the conformational space of an H-NS dimer showed flexible regions in the connectors between the dimerization domains and the DNA-binding domain [6]. The region linking the DNA-binding domain to the N-terminal region of H-NS is shown to be involved in DNA binding [19].

However, the relatively large system size in combination with the slow interaction dynamics in the order of microseconds to seconds, only allows for qualitative predictions, as most of the simulation time is spent with the system being in a stable state. Quantitative predictions, such as the rate constant associated with the binding of H-NS to DNA, require many transitions from one stable state to another. Assuming a binding rate constant in the order of 10^6^ M^−1^ s^−1^, observing a single transition would require at least 1 *μ*s of molecular dynamics simulations. A reasonably accurate estimation of the rate constant, therefore, would require simulation times in the order of seconds, which is currently impossible. Focusing on the transitions regions by avoiding long waiting times in stable states enables a directed end efficient sampling of the transitions [34]. Path sampling techniques achieve this focusing on the transitions by performing an importance sampling procedure in trajectory space [34, 35]. New relevant paths are generated by running relatively short MD trajectories within the transition region, providing a speed up of several orders of magnitude [35, 36]. To compute the reaction rate, we use replica exchange transition interface sampling (RETIS) [35]. The method requires the definition of multiple interfaces λ_*i*_ along the estimated reaction coordinate. For each interface, reactive (from state A to state B) and non-reactive trajectories (from state A back to state A) are collected, from which the probability to reach the next interface can be computed. The product of these probabilities gives the reaction rate [37, 38].

In the first part of this paper, we use all-atom molecular dynamics to characterize the different ways in which the DNA-binding domain of H-NS can bind to a high affinity nucleotide sequence AATATATT based on known H-NS binding sites [3, 10, 24, 28], containing two AT steps. In the second part of the paper, we provide mechanistic insights and a prediction of the rate of the binding of H-NS to a high affinity DNA sequence by means of RETIS simulations.

## Methods

### MD setup

We performed Molecular Dynamics (MD) simulations of the following systems in explicit water: H-NS; dsDNA with nucleotide sequence GCAATATATTGC; and H-NS with GCAATATATTGC. The solution NMR structure of the DNA-binding domain of *Salmonella typhimurium* H-NS-like protein Bv3F (residues 91-139, PDB code 2L93) [16] served as a starting structure for H-NS. As the N-terminal end of this domain is connected to a linker in the full length protein, we placed an acetyl cap on the N-terminus to neutralize its charge. An initial structure for the dsDNA was obtained from the make-na sever developed by Stroud, J. (2006), which models any nucleotide sequence as an ideal B-DNA structure. We chose as a high affinity sequence AATATATT based on known H-NS binding sites [3, 10, 24, 28], Containing two AT steps. This sequence is capped with GC base pairs at both ends to lower the probability of base opening at the DNA ends.

**Fig 1:**
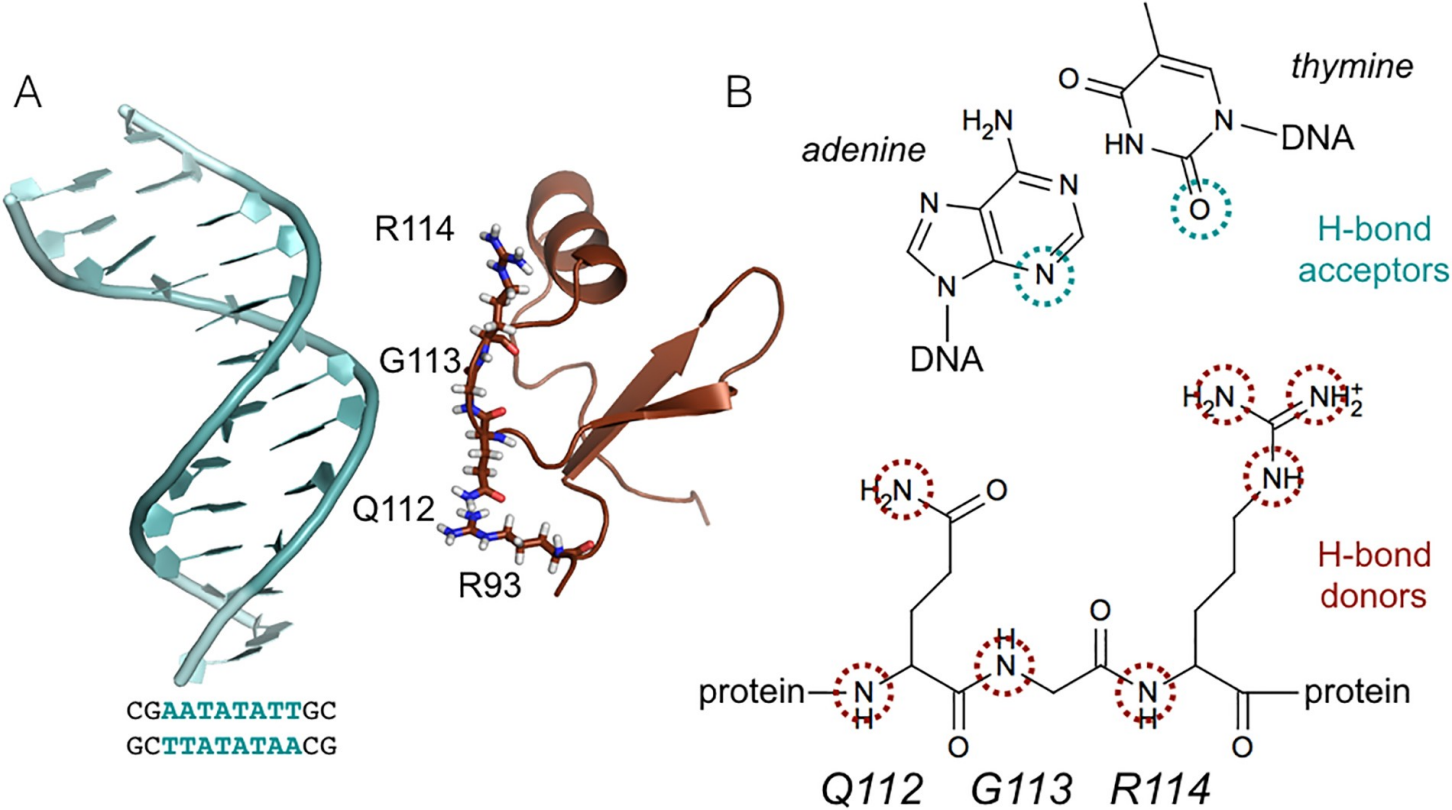
System details. (A) Snapshot of the initial unbound configuration. The protein (brown) and DNA (light blue) are shown in cartoon representation. The QGR motif (residues 112-114) and R93 are shown as sticks. (B) Scheme indicating the hydrogen bond partners involved in the binding of H-NS to DNA. The donors are provided by the QGR motif in H-NS, highlighted by brown circles. The acceptors are located on the bases at the minor groove side of the DNA, indicated by blue circles.

The coordinates of the dsDNA and the C-terminal domain of H-NS were combined resulting in an initial minimum separation distance between H-NS and DNA of 2.0 nm, and are therefore not directly in contact with each other at the start of the MD simulations. Fig 1A shows a snapshot of the initial configuration.

Preparation of the system consisted of placing the structures in a periodic dodecahedron box, with the box boundaries at least 1 nm from the system, followed by the addition of water molecules. To mimic experimental conditions [16] and neutralize the system, we added 50 mM NaCl by replacing water molecules with ions. Interactions between atoms are described by the AMBER03 force field [39] in combination with the TIP3P water model [40]. We selected this particular force field as it contains topologies for both amino acids and nucelotides and performs reasonably well, as long as no major conformational changes are involved [41]. For non-bonded interactions, both van der Waals and electrostatic, we used a cut-off at 0.8 nm. Long range electrostatic interactions were handled by the Particle Mesh Ewald method [42, 43] with a grid spacing of 0.12 nm. To remove unfavorable interactions we performed energy minimization using steepest descents. By applying position restraints on the heavy atoms of the protein and DNA with a force constant in each direction of 1000 kJ/mol nm^2^ and performing 0.1ps of MD at a temperature of 300K and a pressure of 1 bar, we relaxed the water and ions around the initial structures. After preparation, we performed twenty 50 ns MD runs for the H-NS—DNA system, varying initial conditions by assigning new random starting velocities drawn from the Maxwell-Boltzmann distribution at 300K. All simulations were performed with the GROMACS software suite, versions 4.5.3 and 4.5.4 [44, 45] at the Dutch National Supercomputer, Scinet [46], and a locally maintained cluster, with the leap-frog integration scheme and a time step of 2 fs, using LINCS [47] to constrain bonds in the protein and SETTLE [48] to constrain water bonds. All simulations were performed in the isothermal-isobaric ensemble at a pressure of 1 bar, using the v-rescale thermostat [49] and the isotropic Parrinello-Rahman barostat [50, 51].

### Analysis

During the MD simulations, the frames were stored every 20 ps. Analysis consisted of visual inspection using VMD [52] and the calculation of various geometric parameters, including the probability of finding either the protein or the DNA within 0.6 nm of a residue of the DNA or protein respectively, and the probability of finding either Na^+^ or Cl^−^ ions within 0.6 nm of a residue in the protein or DNA. This is achieved by first computing the minimum distance between a given residue and the protein/DNA or ion, and then computing the probability histogram of that distance for all simulations. For the ion probability conformations in either the backbone bound or the fully inserted states were included. In addition we calculated the root mean square deviation (RMSD) of both H-NS and the DNA, with respect to equilibrated starting structures, including all atoms in the calculation. Also, we computed distances and number of hydrogen bonds between hydrogen bond donors in the protein and hydrogen bond acceptors in the DNA. A hydrogen bond is counted when donor and acceptor are within a distance of 0.35 nm and the angle between acceptor, donor and hydrogen is less than 30°. Snapshots are visualized using pymol, developed by Schrödinger, L.L.C. (2010) [53].

A promising quantitative descriptor to follow the interaction between DNA and H-NS has been found in mapping the counting the number of contacts *c*_*ij*_ between hydrogen bond acceptors in the minor groove of DNA, labeled *i*, and hydrogen bond donors in the QGR motif of H-NS, labeled *j*. Fig 1B shows a schematic representation of these hydrogen bond donors and acceptors. For each pair *ij* contacts are counted with the expression:

$$\begin{array}{c} if\;\left(r_{ij}-d_{0}\right)⩽0\;\;c_{ij}=1\;\;\;\;\\ if\;\left(r_{ij}-d_{0}\right)⩾0\;\;c_{ij}=\frac{1-{\left(\frac{\left(r_{ij}-d_{0}\right)}{r_{0}}\right)}^{nn}}{1-{\left(\frac{\left(r_{ij}-d_{0}\right)}{r_{0}}\right)}^{mm}} \end{array}$$ (1)

where *r*_*ij*_ is the distance between atom *i* and atom *j*, located in the DNA and H-NS, respectively. The parameters *r*_0_ = 0.4 nm, *d*_0_ = 0.25 nm, *nn* = 2, *mm* = 4 have been chosen such to count contacts at hydrogen bond distance (< 0.35 nm) as 1 and contacts at 0.7 nm as 0.5. This provides a smooth and descriptive function able to discriminate between the different binding modes. Summing the contacts for all pairs results in the contact map parameter *c*_*QGR*−*minor*_:

$$c_{QGR-minor}=\sum_{j=1}^{N_{{}_{H-NS}}}\sum_{i=1}^{N_{{}_{DNA}}}c_{ij}$$ (2)

where *N*_*DNA*_ and *N*_*H*−*NS*_ are the number of interaction sites in the DNA and in H-NS. In this contact map, hydrogen bond donors in Q112, G113 and R114 have been included. In addition, we calculated the number of contacts between hydrogen bond donors in R93 (atoms N, NZ, NH1 and NH2) and hydrogen bond acceptors in the minor groove of the AT bases *c*_*R*93−*minor*_. To discriminate between different binding modes, the contact map is also computed by considering each hydrogen bond donor in the QGR motif separately with respect to the acceptors in the minor groove of the DNA:

$$c_{j}=\sum_{i=1}^{N_{{}_{DNA}}}c_{ij}$$ (3)

with *j* indicating the atoms Q112-N, Q112-NE2, G113-N, R114-N, R114-NZ, R114-NH1, R114-NH2 in the QGR motif.

### RETIS setup

The computation of the transition rate between two states, labeled *A* and *B*, requires the definition of these states in terms of a set of order parameters. By performing a series of MD simulations, it is possible to characterize the mechanism of the transition between these states and compute its rate. By defining interfaces λ_*i*_ along an order parameter that sufficiently describes the progression of the transition, the transition rate *r*_*AB*_ becomes:

$$r_{AB}=f_{AB}\prod_{i=0}^{N-1}P_{i|i+1}$$ (4)

where *f*_*AB*_ is the flux of MD trajectories passing by interface λ_0_ and *P*_*i*|*i*+1_ are the local probabilities to pass interface λ_*i*+1_ given the crossing of the interface λ_*i*_, called the crossing probability. In essence, the order parameter space is divided in subsections (ensembles) by arbitrarily positioned interfaces. The first ensemble provides the flux, which is a quantification of the frequency of the system escaping stable state *A*. The other ensembles provide the local probability to cross the next interface, given that the previous one has been reached. In regions with large free energy differences, the likelihood to reach the next interface becomes smaller, and therefore a higher number of interfaces is preferable. Further details on the approach, including the mathematical description to compute the flux and the local probabilities, can be found in Refs. [34, 36, 38, 54].

From the MD simulations, we obtained numerical definitions for the stable states, based on the number of contacts between the QGR motif and the minor groove side of the AT basepairs *c*_*GQR*−*minor*_. The BB state is the non-specific interaction between H-NS and the DNA backbone and corresponds to values of 0 < *c*_*GQR*−*minor*_ < 10. The FI state corresponds the full insertion of the Q112, G113 and R114 segment of H-NS, corresponds to values of *c*_*QGR*−*minor*_ > 30. An intermediate meta-stable state, state PI, has been also detected for values of *c*_*QGR*−*minor*_ around 20, which corresponds to an insertion of either Q112 or R114 into the minor groove. According to the RETIS procedure and following the developers’ guidelines [36, 38, 54], interfaces have been positioned along the order parameter. If during the preparatory procedures steep gradients were observed, more interfaces were added. In other words, if the probability to cross the next interface from a particular interface becomes very low (less than 20% as a rule of thumb), an additional interface should be added. Setting up a RETIS simulation is a trade-off between keeping the number of interfaces as low as possible, to reduce computational costs, and maintaining sufficient crossing. It is worthwhile to point out that an optimal interface number and positioning would improve the efficiency of the sampling, but it would not affect the final results. In cases where the potential energy surfaces is as complex as in the present study, it is computationally exceedingly expensive to achieve the optimal set up. In the binding of H-NS to DNA, a metastable state PI has been identified which imposed a division of the sampling of the overall transition in two regions: BB → PI and PI → FI. In the BB → PI transition, the interfaces has been located at values for *c*_*QGR*−*minor*_ as follows: $\lambda_{0}^{BB-PI}=10$, $\lambda_{1}^{BB-PI}=12$, $\lambda_{2}^{BB-PI}=14$, $\lambda_{3}^{BB-PI}=16$, $\lambda_{4}^{BB-PI}=18$, $\lambda_{5}^{BB-PI}=20$. For the PI → FI transition the interfaces are located at values for *c*_*GQR*−*minor*_: $\lambda_{0}^{PI-FI}=20$, $\lambda_{1}^{PI-FI}=22$, $\lambda_{2}^{PI-FI}=24$, $\lambda_{2}^{PI-FI}=26$, $\lambda_{2}^{PI-FI}=28$, $\lambda_{3}^{PI-FI}=30$. Several cycles of RETIS simulations allowed for an educated guess of the interface positions.

A series of snapshots have been randomly selected from a regular MD trajectory connecting the *BB* and *FI* states, covering the range of value of *c*_*QGR*−*minor*_ between BB and PI and PI and FI. These trajectory fragments constituted the initial points to start the RETIS simulations. The RETIS simulations have been performed via the PyRETIS library [37].

RETIS considers three Monte Carlo based moves. Two way shooting, time reversal and swapping. A two-way shooting move consists of starting two MD simulations from a randomly selected snapshot on the previously accepted trajectory. The shooting points, to which random velocities have been assigned respecting the Boltzmann distribution of kinetic energies for the given temperature (aimless shooting), are the seeds from which the new trajectories are propagated along positive and negative, times to generate a full new trajectory. Two consecutive trajectories have, in this scheme, only one point in common. In time reversal moves the trajectory is reversed in time by reversing the sequence of snapshots and by reversing the velocities of each atom in each snapshot. In the swapping moves, two trajectories that both satisfy the properties of both relative ensemble are swapped. A description of the moves and the criteria to select their relative frequency is provided in Refs. [38, 54].

Initially, to decorrelate the paths with the initials snapshots, a total of about 400 two-way aimless shootings moves have been performed in 4 independent parallel simulations. Thereafter, the production runs consisted in in about 500 RETIS cycles for the rate estimation of the transitions BB → PI and PI → FI. A cycle corresponds to a RETIS move for each ensemble. For 12 ensembles, a total of 6000 trajectories have been considered of which 1500 generated by shooting moves, 1500 by time-reversal moves and the remaining by swapping moves [36, 38]. The resulting acceptance path ratio has been equal to 0.31, while the average path length for BBtoPI and PItoFI has been around 30000 time steps corresponding to 60 ps. The aggregate simulation time is around 200 ns. For each simulation, the order parameter and the other descriptors have been stored every 0.1 ps.

To visualize the underling mechanisms, two-dimensional histograms of the path ensembles of the transitions BB → PI and PI → FI have been produced, projected onto pairs of selected descriptors. From all the generated paths, the relative frequency of visiting a 2D region has been computed in a grid of 500 by 500 bins which subdivided the interval between the minimum and maximum value of each respective descriptor.

## Results

### Different modes of binding

**Fig 2:**
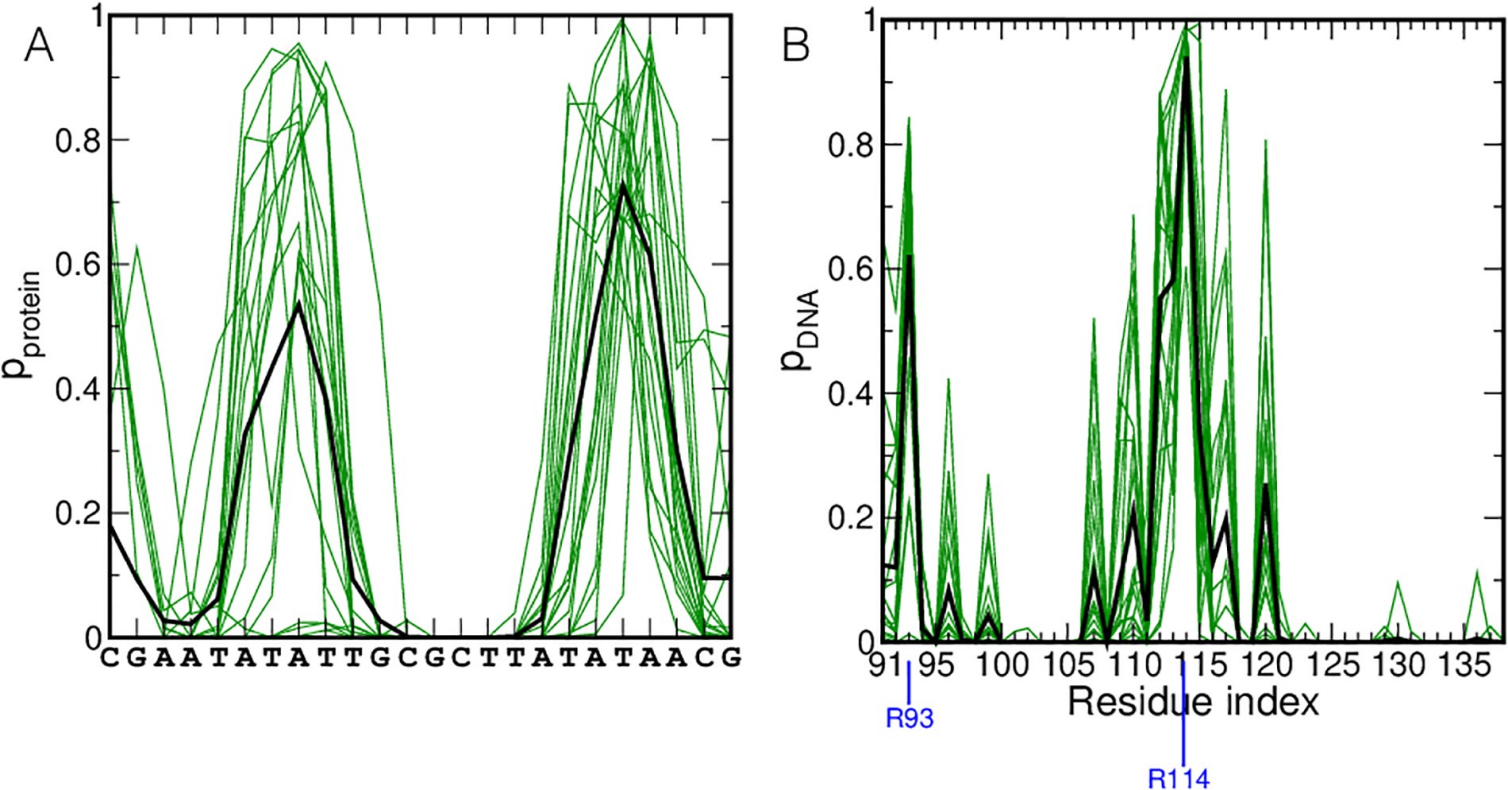
Proximity of H-NS and DNA. (A) The probability of finding the protein within a minimum distance of 0.6 nm of the DNA is plotted as a function of the nucleotide sequence. (B) The probability of finding the DNA within a minimum distance of 0.6 nm of the protein is plotted as a function of the amino acid sequence. The green lines indicate the probabilities for the individual trajectories, the black line indicates the average over all trajectories. Residues R93 and R114 are marked with a blue line.

Several studies have indicated that H-NS has a preference for AT-rich DNA, [25, 26, 28, 29]. Additional research has shown that H-NS binds DNA with a highly conserved motif consisting of three amino acids, QGR, in the DNA-binding domain [4, 17]. To confirm these observations with molecular dynamics, we performed twenty 50 ns MD simulations of the DNA binding domain of H-NS and an AT-rich nucleotide sequence. For the latter, we selected AATATATT, capped with GC, resulting in sequence 5’-GCAATATATTGC-3’, based on known H-NS binding sites [3, 10, 24, 28] For the part of H-NS, we used the NMR structure for residues 91-139 (PDB code 2L93 [16]). The protein and the DNA were placed at a separation distance of 2 nm, see Fig 1A. Note that no hydrogen bonds are formed between the protein and the DNA at the start of the MD simulations. We have conducted MD simulations with a larger distance of separation between the protein and the DNA. These simulations resulted in many complexes with the protein attached to one of the DNA ends. Such conformations are not relevant from a physiological point of view. Visual inspection of the MD trajectories reveals that H-NS binds to the DNA in all trajectories. The protein has a strong preference for binding to the AT region in the DNA sequence, as evidenced by the high probabilities for finding protein within 0.6 nm of a nucleotide, see Fig 2A. The probabilities at the CG ends of the DNA arise from the protein binding to the DNA end. Fig 2B shows the probability of finding DNA within 0.6 nm of a protein residue. The highest probability occurs for R114, followed by G113, Q112 and R93. These residues are all conserved and when altered, abolish DNA binding [16, 19].

**Fig 3:**
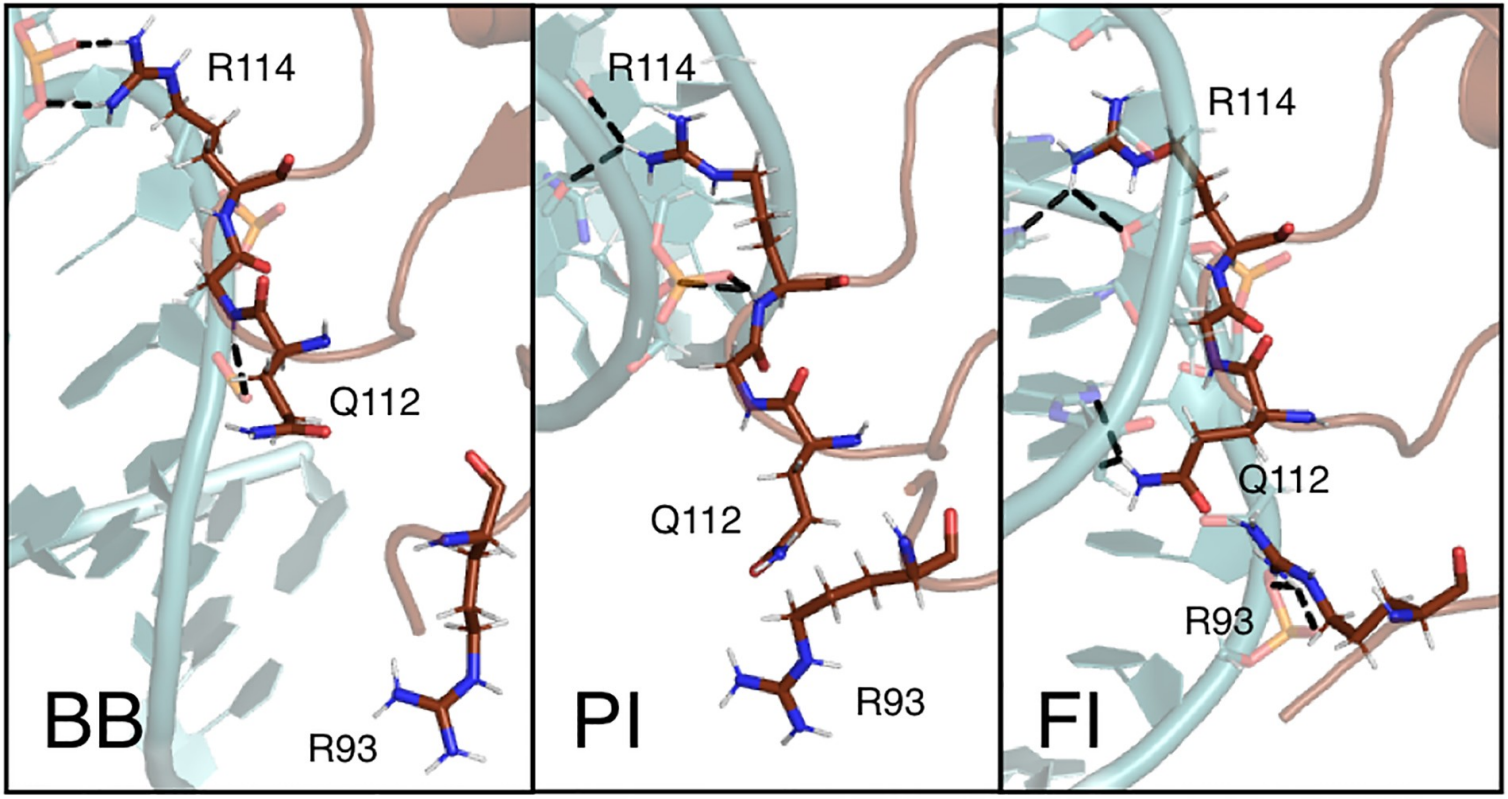
Snapshots of the modes of interaction between H-NS and DNA. The protein (brown) and DNA (light blue) are shown in cartoon representation. The QGR motif and R93 are shown as sticks. The labels indicate the different binding modes: (BB) Backbone bound, (PI) Partial insertion and (FI) full insertion.

For the hydrogen bond donors in the residues identified as interacting with DNA (R93, Q112, G113 and R114) we have plotted time traces of the minimum distance between the hydrogen bond donors in these residues and hydrogen bond acceptors in the minor groove of the AT basepairs $d_{DNA-N}^{min}$. These time traces are shown in the Supporting Information, S1 Fig. Three main H-NS binding categories can be identified, based on interactions of the QGR motif with the minor groove of the DNA: bound to the DNA backbone (BB), with one side chain of the QGR motif inside the minor groove, referred to as partially inserted (PI) and with the entire QGR motif inside the minor groove, referred to as fully inserted (FI). Fig 3 shows snapshots of these three modes. A movie of one of the trajectories showing H-NS binding to DNA and visiting the three modes is given in the Supporting Material, S1 and S2 Movies. For each run in S1 Fig we indicated in which state the MD simulation ended. The time traces S1A Fig, columns Q112NE2, R114NH1 and R114NH2, show that partial insertion can occur with the sidechain of either Q112 or R114 (indicated as PI-Q and PI-R respectively). The distance between Q112 and th DNA fluctuates more than the distance, between R114 and the DNA, suggesting that the former may be less stable. As these MD simulations have not sampled all states exhaustively, we refrain from making more quantitative statements based on this data. Fig 2 suggested a role for R93 in the binding of DNA. The time traces of the individual interactions of all hydrogen bond donors in this residue with the minor groove indicate that this residue indeed interacts with DNA, but to a lesser extent than the GQR motif.

**Fig 4:**
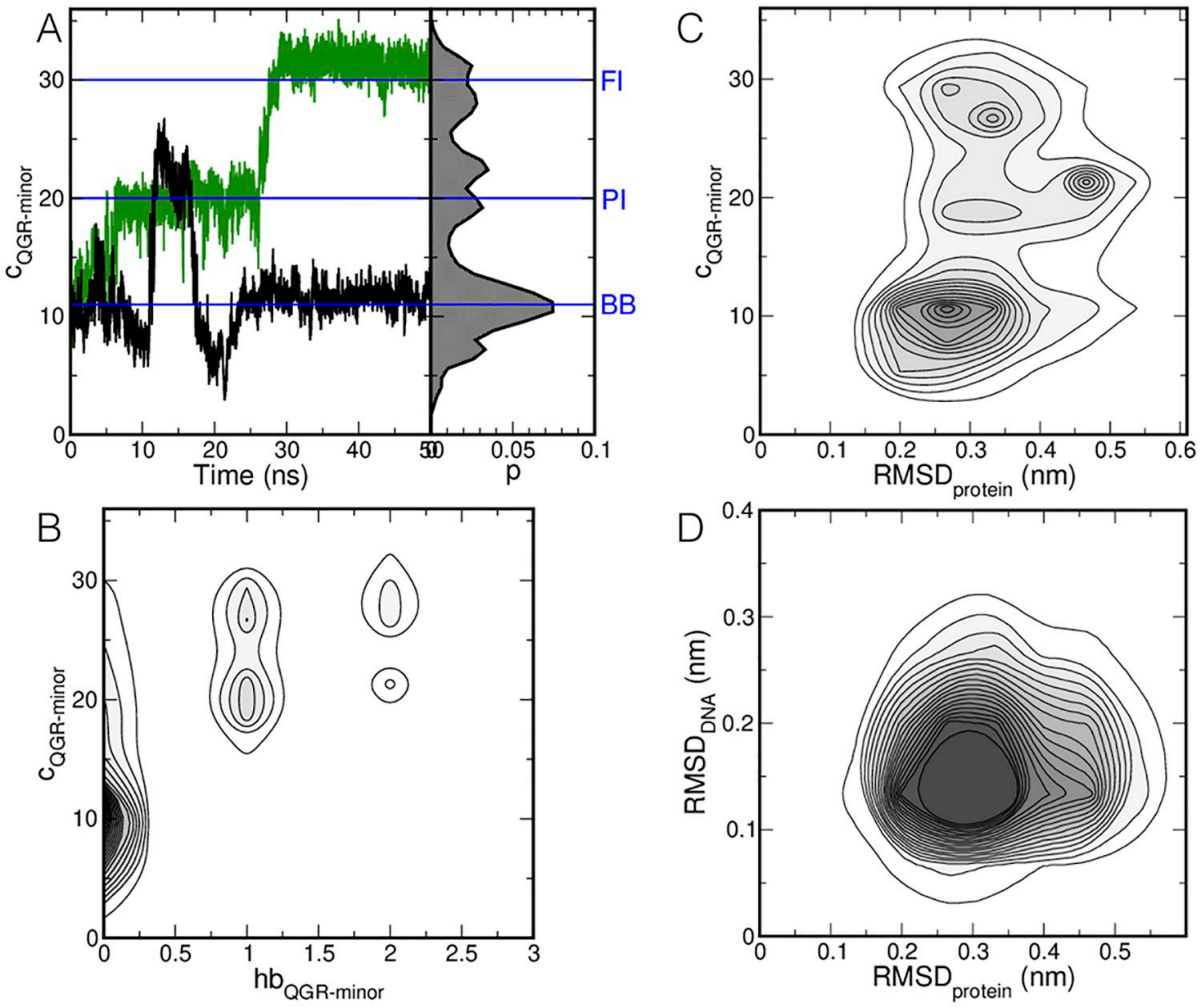
Results of the MD simulations. (A) The number of contacts between the hydrogen bond donors in the QGR motif and hydrogen bond acceptors in the minor groove of the AT basepairs in the DNA *c*_*QGR*−*minor*_ is plotted as a function of time for two representative runs (left), and given as a probability histogram over all MD simulations (right). (B) Probability density plot of the number of hydrogen bonds in the QGR motif and the minor groove of the AT basepairs and *c*_*QGR*−*minor*_. (C) Probability density as a function of the protein RMSD and *c*_*QGR*−*minor*_. (D) Probability density as a function of the protein RMSD and the DNA RMSD. Darker colors indicate a higher probability. Contour lines are drawn every 0.05%.

The three interaction modes differ in number of contacts between the QGR motif and the DNA. To quantify the number of contacts and establish that the states identified by visual inspection are indeed stable states, we computed the number of contacts *c*_*QGR*−*minor*_ between the hydrogen bond donors in the QGR motif and hydrogen bond acceptors in the minor groove for the AT base pairs, see Fig 1B for a schematic drawing of the number of contacts. The time traces shown in Fig 4A represent two trajectories in which all binding modes are visited. Data from all 20 simulations is collected in a probability histogram of *c*_*QGR*−*minor*_ in Fig 4A, indicating the three states. The backbone bound state occurs at *c*_*QGR*−*minor*_ around 10, followed by the partial inserted binding mode at *c*_*QGR*−*minor*_ around 20. The fully inserted binding mode occurs at *c*_*QGR*−*minor*_ larger than 30. We chose the descriptor *c*_*QGR*−*minor*_ to distinguish the different binding modes over the number of hydrogen bonds, as *c*_*QGR*−*minor*_ allows for a clearer separation of the different binding modes. This aspect is shown in Fig 4B as a probability density plot of the number of hydrogen bonds between the QGR motif and the minor groove of the DNA *hb*_*GQR*−*minor*_ and *c*_*QGR*−*minor*_. The number of hydrogen bonds increases with *c*_*QGR*−*minor*_, but does not distinguish clearly between the different binding modes.

Expanding the probability histogram into a second dimension describing the RMSD of the protein with respect to the starting configuration, see Fig 4C, shows that the RMSD does not exceed 0.4 nm, indicating that the conformational fluctuations in the protein involve side chain re-orientation and that the overall structure of the protein changes little upon binding to DNA. Similarly, as the RMSD of the DNA remains below 0.4 nm, see Fig 4D, no large conformational changes occur upon binding H-NS.

When H-NS is in the BB state, the QGR motif interacts with either the phosphate and/or the deoxyribose groups, see Fig 3. R114 can form multiple salt bridges with the phosphate groups, whereas Q112 and G113 can form hydrogen bonds with the DNA backbone. In the BB binding mode, the QGR motif can adopt many different orientations with respect to the DNA backbone, some of which also involve R93. In the PI binding mode, one residue of the QGR motif interacts with one or more bases in the minor groove of the DNA via hydrogen bonds. Partial insertion can occur with either R114 or with Q112, see Fig 3. When R114 is inside the minor groove, Q112 interacts with either the phosphate backbone, R93 or solvent. R114 interacts with phosphate groups when Q112 is inside the minor groove. The protein typically covers one to four base pairs when partially inserted, depending on the orientation of residues in the QGR motif not involved in minor groove interactions. Finally, full insertion of the QGR motif occurs when both Q112 and R114 form hydrogen bonds with bases in the minor groove, see Fig 3. When fully inserted, the QGR motif is aligned with the phosphate backbone and covers three to four base pairs. G113 can also form hydrogen bonds to the bases, causing Q112 and R114 to extend such that the QGR motif covers five base pairs in the minor groove.

**Fig 5:**
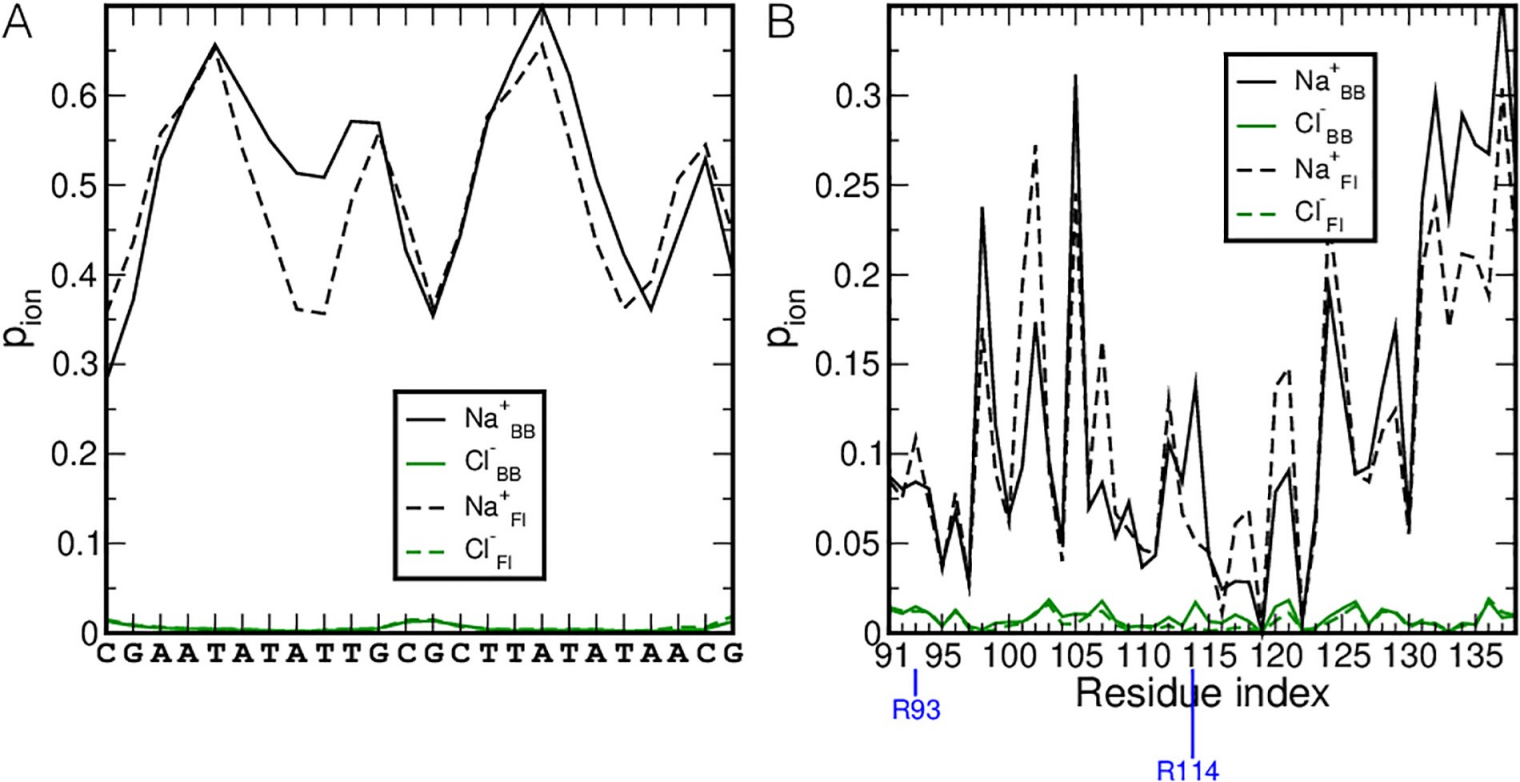
Proximity of ions during binding. The probability of finding Na^+^ (black lines) and Cl^−^ (green lines) within a minimum distance of 0.6 nm of (A) the DNA and (B) the protein is plotted as a function of (A) the nucleotide sequence or (B) the amino acid sequence. The dotted and solid lines indicate the probabilities for the BB and the FI states respectively. Residues R93 and R114 are marked with a blue line.

Finally, we investigated whether the location of ions on the DNA and protein change upon binding. To this end we calculated the probability of finding a Na^+^ or Cl^−^ ion close to the protein or DNA, *p*_*ion*_, see Fig 5. For the DNA we observe that less Na^+^ ions are located close to the TATT bases in the FI binding mode compared to the BB state. For the protein, changes in *p*_*ion*_ occur for both Na^+^ and Cl^−^ in going form BB to FI. The drop in finding ions close to R114 clearly relates to its insertion into the minor groove. Increases in *p*_*ion*_ for sodium may indicate that in the FI state those residues have lost the transient interactions with R93 or R114, which are now replaced by Na^+^. The decrease of sodium interactions in the FI state for residues 130-139 may be a consequence of the DNA being closer to those residues.

### Mechanism and rates of H-NS binding to DNA

**Fig 6:**
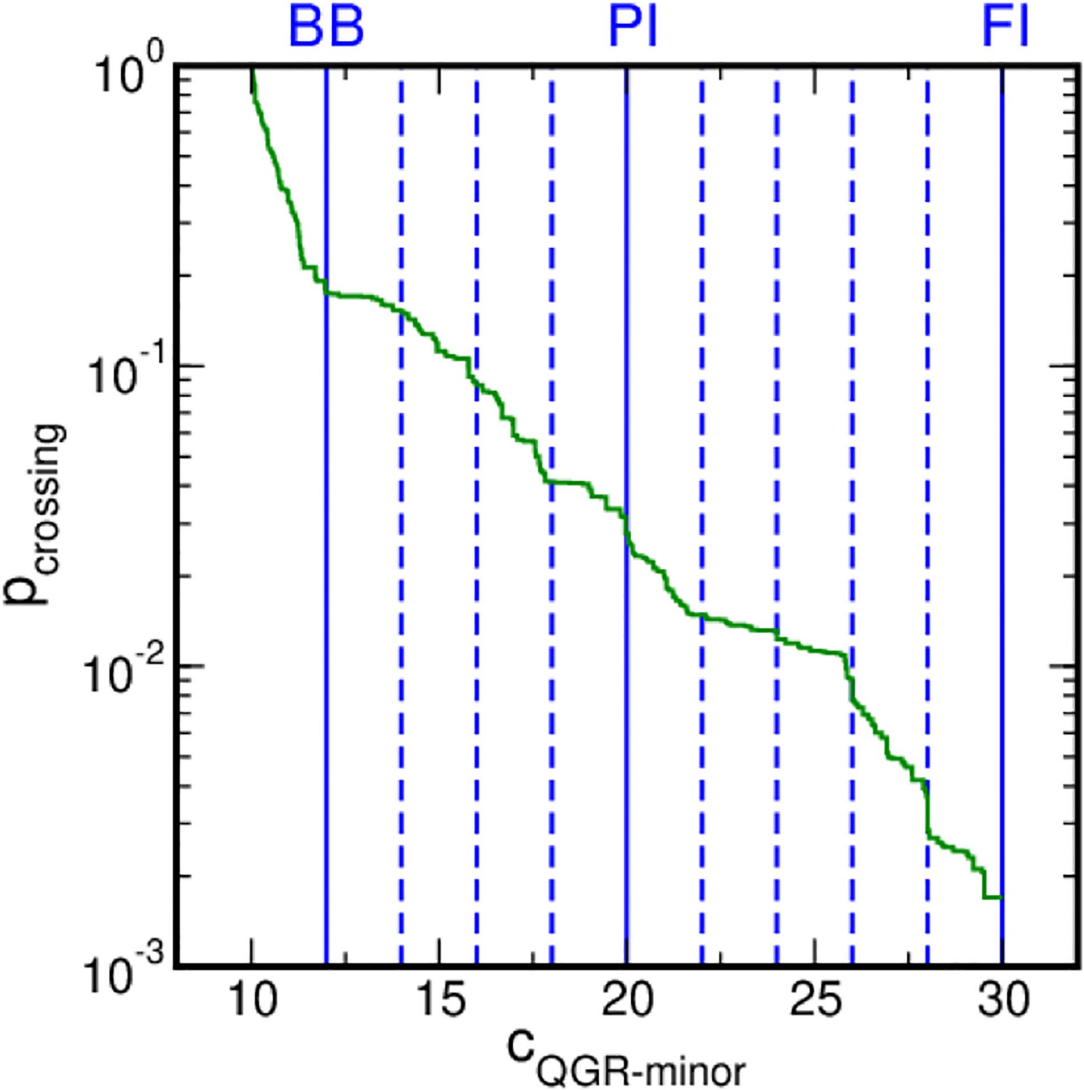
Crossing probabilities in the RETIS simulations. The crossing probability is shown as a function of *c*_*QGR*−*minor*_ in green. The profile quantifies the probability for a path starting from the BB state to reach a certain *c*_*QGR*−*minor*_ value. The blue lines indicate the location of the interfaces. The solid lines indicate the location of the stable states.

Upon binding to AT-rich DNA, the QGR motif visits various stable states with a residence time of more than 10 ns. We performed RETIS simulations to characterize the mechanisms of the transitions between these states. Starting from an initial trajectory that samples a transition, RETIS collects trajectories along the order parameter space by monitoring MD simulations [37]. Once a simulation enters a stable state, it is stopped, limiting an excessive sampling of the stable state. RETIS calculations thus permits to compute the rate constant for H-NS binding to DNA. The MD simulations show that the number of contacts between the QGR motif and the minor groove side of the AT basepairs in the DNA *c*_*QGR*−*minor*_ sufficiently indicates the progression of H-NS binding to DNA. Fig 6 reports the results of the weighted histogram analysis method applied to the local crossing probabilities as a function of the main order parameter *c*_*QGR*−*minor*_ for the BB to PI to FI transitions. The profile of the combined crossing probabilities is smooth, indicating that *c*_*GQR*−*minor*_ is a good choice as a reaction coordinate to describe the binding of H-NS to DNA and that no additional processes play a role.

Table 1 reports the values of the flux, the crossing probabilities and the resulting rate constants, and relative errors. No paths that directly connect states BB and FI have been sampled. This suggests that full insertion of the QGR motif occurs via an intermediate state in which the motif is partially inserted. Since BB and PI transitions are adjacent in the considered order parameter space, an overall rate *r*_*BBtoPI*_ can be computed by multiplying the crossing probabilities *P*_*BBtoPI*_ and *P*_*PItoFI*_ by the flux out of the BB state *ϕ*_*BB*_. The rates for the three transitions BB to PI, PI to FI and BB to FI are given in Table 1. The errors on the estimation of the rate for transition events in complex energy lanscapes is commonly accepted to be within an order of magnitude [55]. We therefore consider the 100% error achieved to be relatively modest and within the accuracy of the force field. Further computations would, therefore, add only a very limited value without providing new insight into the process.

**Table 1:** Results from the RETIS simulations.

Transition	flux s^−1^	*P*_*cross*_	rate s^−1^
BB to PI	2.57 ⋅ 10^9^ ± 39%	2.78 ⋅ 10^−2^ ± 70%	7.15 ⋅ 10^7^ ± 88%
PI to FI	2.39 ⋅ 10^9^ ± 17%	6.14 ⋅ 10^−2^ ± 47%	1.48 ⋅ 10^8^ ± 51%
BB to PI to FI	2.57 ⋅ 10^9^ ± 39%	1.70 ⋅ 10^−3^ ± 85%	4.38 ⋅ 10^6^ ± 93%

The flux indicates the frequency of escaping the initial state, the crossing probability is the probability of reaching the next interface when crossing interface *i*. The reaction rate is calculated according to Eq 4. All values are given with their relative errors, obtained by dividing the standard deviation by the square root of the sample size.

After non-specific association to the backbone, the insertion of H-NS into the minor groove occurs in the order of 10^6^ M^−1^ s^−1^. When considering the binding of H-NS to DNA as limited by diffusion, the forward rate of the process is in the order of 10^8^ M^−1^s^−1^ [56]. Including electrostatic consideration would make the non-specific association even faster [56]. We therefore conclude that the transition from BB to the fully inserted conformation is the rate-limiting step in the binding process of H-NS to DNA.

**Fig 7:**
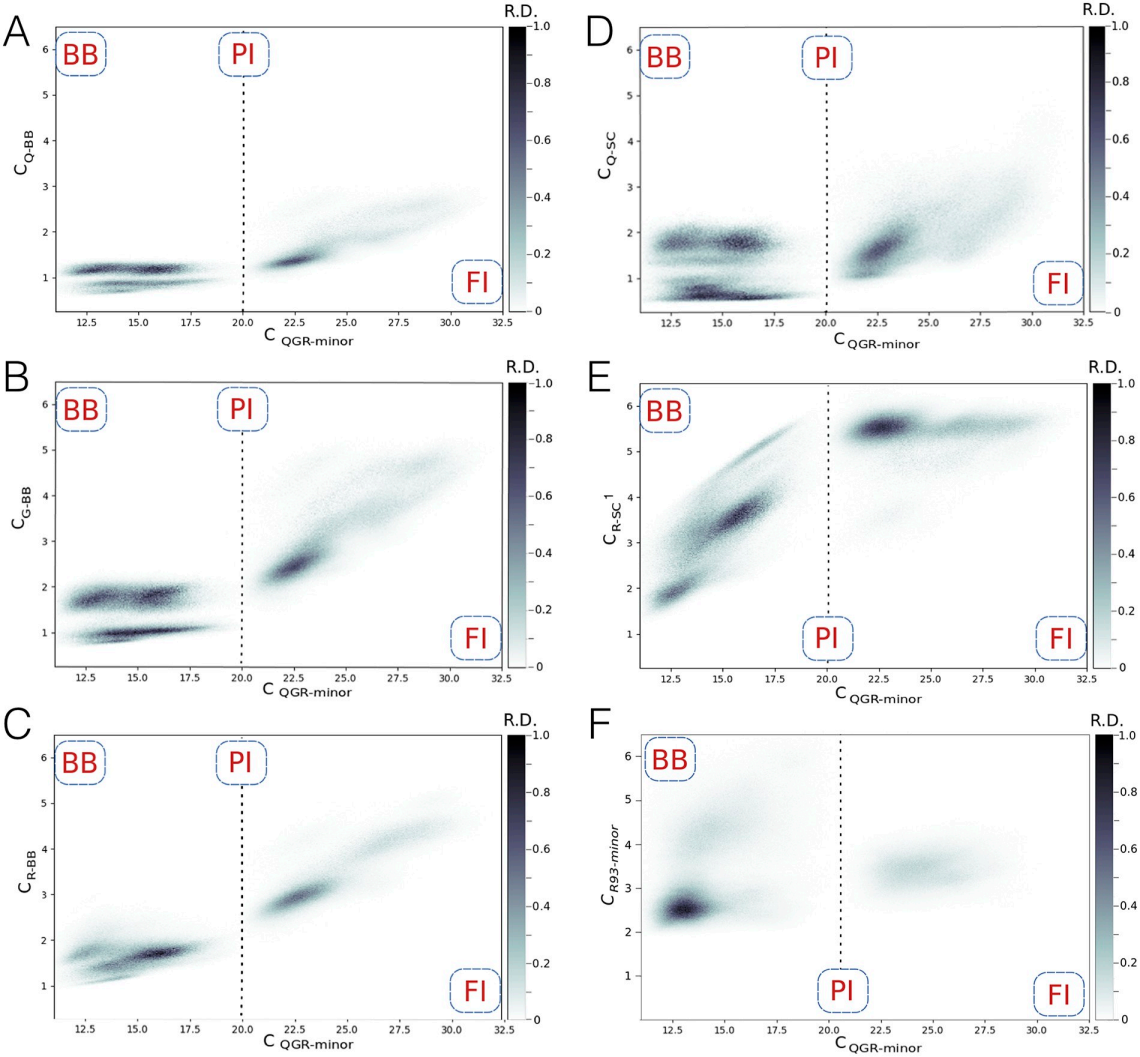
Probability density plots of the RETIS trajectories for the BB to PI to FI transition. The horizontal axis represents the number of contacts between hydrogen bond donors in the QGR motif and hydrogen bond acceptors at the minor groove side of the AT basepairs *c*_*QGR*−*minor*_. The vertical axis represents a local number of contacts between the minor groove side of the AT basepair and (A) the backbone N of Q112 *c*_*Q*−*BB*_, (B) the backbone N of G113 *c*_*G*−*BB*_, (C) the backbone N of R114 *c*_*R*−*BB*_, (D) the side chain NE2 of Q112 *c*_*Q*−*SC*_, (E) the side chain NH1 of R114 *c*_*R*−*SC*1_ and (F) the hydrogen bond donors N, NZ, NH1 and NH2 in R93 *c*_*R*93−*minor*_. The labels indicate the location of the states on the horizontal axis. The dashed line indicates the location of the PI state. The color indicates the relative density. Darker regions are more often visited. White areas have not been sampled.

By projecting the trajectories collected during the RETIS simulations onto relevant geometrical parameters allows for insights into the mechanism in the form of a probability density plot. Fig 7 shows the RETIS trajectories as a probability density projected onto *c*_*QGR*−*minor*_ and several number of contact counts between the minor groove of the AT basepairs and the individual hydrogen bond donors in the QGR motif. In all trajectories the number of contacts between the hydrogen bond donors in the Arg114 sidechain (atom NH1) and the DNA start to increase from 2 to 5 contacts before forming the PI state at *c*_*QGR*−*minor*_ = 20 (Fig 7E), while the other hydrogen bond donors do not form increasingly more contacts This observation indicates that the sidechain of R114 is the first to enter the minor groove to form the PI state. When comparing the profiles focused on the protein backbone contacts, Fig 7(A)–7(C), the backbones of R114 and G113 insert before Q112. The sidechain of Q112 is the last to enter the minor groove, which happens after *c*_*QGR*−*minor*_ = 23. We also computed the number of contacts between the hydrogen bond donors in R93 and the minor groove *c*_*R*93−*minor*_ and plotted this as a probability density in Fig 7(F). The number of contacts in the overall transition from BB to FI averaged to around 3, indicating interactions between R93 and the minor groove. In the transition from BB to PI two density spots appear at *c*_*R*93−*minor*_ = [2, 3] and *c*_*R*93−*minor*_ = 4, suggesting that R93 has multiple ways of interacting with the DNA. The calculation of *c*_*R*93−*minor*_ involves counting the contacts between four atoms in R93 and the minor groove and can be qualitatively compared to *c*_*QGR*−*minor*_, which is dominated by the number of contacts between R114 and the minor groove. Compared as such, the values for *c*_*R*93−*minor*_ are low, indicating that R93 does not insert in the minor groove, but rather interacts with the DNA backbone. Summarizing, binding of the H-NS DNA binding domain to the minor groove of AT basepairs occurs via the QGR motif, and also involves R93.

**Fig 8:**
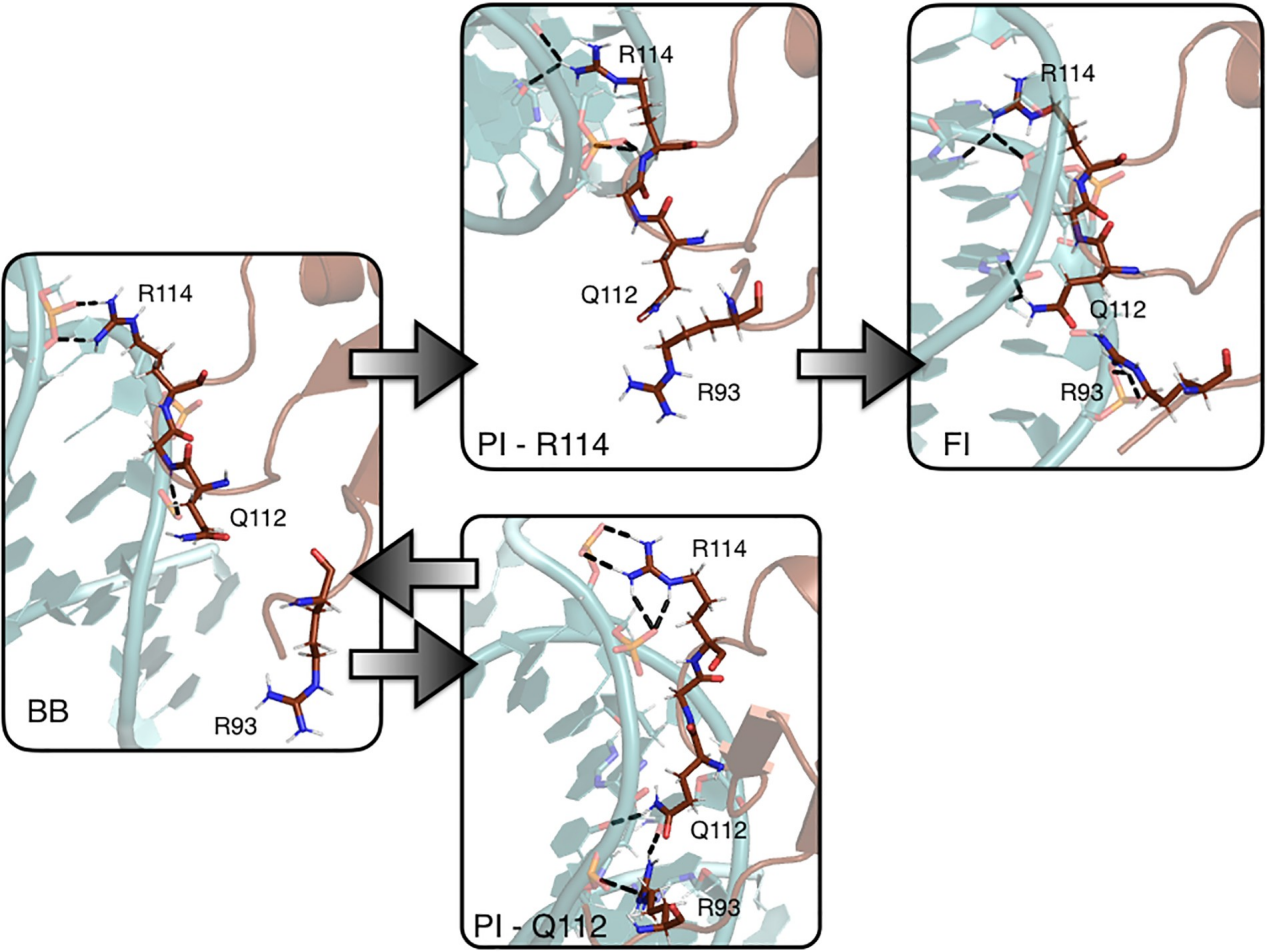
Mechanism of H-NS binding to AT-rich DNA. Snapshots of the modes of interaction between H-NS and DNA. The protein (brown) and DNA (light blue) are shown in cartoon representation. The QGR motif and R93 are shown as sticks. The arrows indicate in which direction the transitions occurred in the RETIS simulations.

Our observations from both the MD and RETIS simulations suggest a sequential mechanism for the insertion of the QGR motif into the minor groove, initiated by the side chain of R114, as summarized in Fig 8. The time traces obtained from the MD simulations in S1 Fig all show that the FI state is formed via insertion of the sidechain of R114 followed by insertion of the sidechain of Q112. These observations are confirmed in the RETIS simulations. The MD simulations occasionally show the formation of a PI state with Q112 inserted. This interaction fluctuates more than the interaction between R114 and the minor groove. Furthermore, the time traces show that insertion of Q112 can be followed by a return to the BB state, which is also confirmed by the RETIS simulations.

## Discussion

In this work, we show that the conserved QGR motif is the main initiator of DNA binding by H-NS, aided by another arginine, R93. In our simulations we did not observe any other interactions between H-NS and the DNA that lasted longer than 10ns. These findings agree with the experimental results by Gordon *et al*., who found that H-NS binding is mediated by a Q/RGR motif [16]. The role of R93 emphasizes the recently identified importance of charged residues in the linker region, as recently found by superresolution microscopy studies on the DNA binding properties of H-NS mutants [19]. H-NS can bind to DNA in various modes, which we grouped into three categories: bound to the backbone (BB), the QGR motif partially inserted into the minor groove (PI) and fully inserted into the minor groove (FI). Our results show that a fully inserted QGR motif has a lifetime of more than 50ns. Moreover, this complex is structurally in excellent agreement with the docked structure proposed by Gordon *et al*. [16].

Currently more accurate force fields are available for both proteins [57] and nucleic acids [58]. Despite using a less accurate force field, AMBER03 [39], we were able to reproduce experimental observations, and provide additional insights into the binding process. These results did not involve any major conformational changes in the protein nor in the DNA, and therefore, we believe that the AMBER03 force field proved to be sufficient.

The MD simulations indicate that the number of base pairs shielded by the QGR motif during complex formation depends on the orientation and conformation of the QGR motif when bound to DNA. Root mean square fluctuation (RMSF) calculations of the DNA-binding domain with respect to the NMR structure, using C-alpha atoms, indicate that the QGR motif is one of the more flexible parts of the domain (data not shown). The motif exhibits various conformations ranging from the two side chains (Q112 and R114) fully extending along the principal axis of the protein backbone or curving away from the backbone. This flexibility enables the QGR motif to cover three to five base pairs, while aligned along the phosphate backbone. In the FI state three to five base pairs are covered and one to four base pairs are shielded by H-NS in the PI binding mode. When bound to DNA, the H-NS DNA-binding domain prevents additional domains to bind directly adjacent to the first. This excluded volume effect shields up to three extra base pairs in addition to the base pairs directly occluded by the QGR motif. Cross-linking experimental studies have identified the size of the binding site of H-NS between 15-20 base pairs [59], which was further refined to 8 to 10 base pairs based on NMR experiments experiments combined with an bioinformatics approach [60]. These observations agree well with our predictions.

The models of a full-length H-NS dimer as constructed by van der Valk *et al*. [61] allows for an interpretation of our results in a larger context. These models were constructed by combining the structural information for the N-terminal oligomerization domain (PDB:3NR7 [14]) and the NMR structure of the DNA-binding domain (PDB:2L93 [16]) with the flexible linker modeled as a random coil [61]. The distance between two DNA-binding domains in these models depends on the distances between the C-terminal ends of the oligomerization domains, the conformation of the linker region and the conformation of the DNA-binding domain. Since the RMSD of the DNA binding domain indicates that hardly any structural changes occur upon binding (see Fig 4D, we measured the distance between the N-terminal end of the DNA binding domain (A91-C) and the center of the QGR motif (G113-CA) at 1.9 nm. The linker is 1.2 nm in length when in random coil conformation and 2.4 nm when fully stretched. Finally, the distance between the C-terminal ends of the oligomerization domains is 9 nm when dimerization occurs via the N-terminal dimerization site and 2.3 nm when dimerization occurs via the dimer-dimer interaction site. As a first estimate for the range of distances between two DNA-binding domains in a H-NS filament on DNA, we estimate that the distance between adjacent QGR motifs ranges from 8 to 17 nm. However, as more recent MD simulations indicate considerable flexibility for both the helix connecting the two dimerization domains in the oligomerization domain [6] and the region linking the DNA-binding domain to the rest of the protein [6, 19], this interpretation is prelimiary, and warrants further modeling, but this is beyond the scope of this work.

A recent bioinformatics study revealed that arginine residues have a preference for narrow minor grooves [62]. This study suggested that arginine binding to narrow minor grooves is a mechanism widely used in protein-DNA recognition. Electrostatic potential calculations showed that narrow minor grooves have a strongly enhanced negative electrostatic potential, providing an explanation for the preference of arginine to bind to narrow minor grooves [62]. Glutamine was identified as the third most frequent narrow minor groove binder, following arginine and lysine. These results agree well with the prominent role of R114 in the H-NS DNA binding modes and suggest that the QGR motif is optimal for binding to narrow minor grooves.

As lysine was identified as a frequent minor groove binder, we investigated whether lysine residues in the H-NS DNA-binding domain contributed to DNA binding. The DNA-binding domain contains six lysine residues, of which four may interact with the DNA via salt bridge interactions to phosphate groups, see Fig 2 Interactions of lysines with the bases from either the minor or major groove of the DNA are not observed. Electrostatic potential calculations indicated that the energetic cost of desolvation is larger for lysine than for arginine [62]. We speculate that the lysines in the H-NS DNA binding domain may provide additional attractive interactions during the initial stage of H-NS moving closer to the DNA.

Different studies suggest that H-NS favors curved DNA over straight DNA [23, 63, 64]. Since twelve base pair DNA is too short to determine curvature, we cannot comment on the link between the various binding modes and the curvature of the nucleotide sequences. However, since the QGR motif only binds a short region of DNA, we believe it to be unlikely that individual DNA-binding domains discriminate between curved and straight DNA.

### Conclusion

Molecular dynamics simulations revealed that the H-NS DNA-binding domain binds to DNA with its conserved QGR motif, aided by Arg93. The association of the protein domain to DNA does not result in deformation of either the protein or the DNA. We identified three binding modes, which can be distinguished via the number of contacts between the QGR motif and the DNA. The binding modes, in order of increasing number of contacts between the protein and the bases, are BB (backbone bound) PI (partially inserted) and FI (fully inserted). Replica Exchange Ttransition Interface Simulations enabled the calculation of the rate of the transition from the backbone bound state to the fully inserted state. These calculations indicate that the fully inserted state is always reached via the partially inserted intermediate, and that R114 initiates the binding. The rate of going from BB to FI is predicted to be in the order of 10^6^ M^−1^ s^−1^.

## Supporting information

S1 FigTime traces of the hydrogen bond interactions between H-NS and DNA.The minimum distances between hydrogen bond acceptors in the minor groove of the AT basepairs and hydrogen donors in protein residues $d_{DNA-N}^{min}$ are plotted for each MD run and for each donor, for (A) Q112-N, Q112-NE2, G113-N, R114-N, R114-NZ, R114-NH1, R114-NH2 and (B) R93-N, R93-NZ, R93-NH1, R93-NH2. The labels at the righthand side of the graphs indicate the state observed at the end of each 50 ns run. The red line in each graph indicates the distance cutoff for a hydrogen bond interaction, at 0.35 nm. The star indicates which runs have been plotted in Fig 4(A).(TIF)

S1 Movie Movie of the binding of the H-NS DNA-binding domain to DNA. The protein (brown) and DNA (light blue) are shown in cartoon representation. The QGR motif and R93 are shown as sticks. The orientation is from the top of the DNA. Note that this movie shows the same trajectory as S2 Movie.
(MP4)

S2 Movie Movie of the binding of the H-NS DNA-binding domain to DNA. The protein (brown) and DNA (light blue) are shown in cartoon representation. The QGR motif and R93 are shown as sticks. The orientation is from the side of the DNA. Note that this movie shows the same trajectory as S1 Movie.
(MP4)

## References

[1] Falconi M, Gualtierl M, Teana A, Losso M, Pon C (1988) Proteins from the prokaryotic nucleoid: primary and quaternary structure of the 15-kD Escherichia coli DNA binding protein H-NS. Molecular microbiology. 2(3):323–329. 10.1111/j.1365-2958.1988.tb00035.x 3135462

[2] Williams RM, Rimsky S (1997) Molecular aspects of the E. coli nucleoid protein, H-NS: a central controller of gene regulatory networks. FEMS microbiology letters. 156(2):175–185. 10.1111/j.1574-6968.1997.tb12724.x 9513262

[3] Dame RT, Wyman C, Goosen N (2000) H-NS mediated compaction of DNA visualised by atomic force microscopy. Nucleic acids research. 28(18):3504–3510. 10.1093/nar/28.18.3504 10982869 PMC110753

[4] Dorman CJ (2004) H-NS: a universal regulator for a dynamic genome. Nature Reviews Microbiology. 2(5):391–400. 10.1038/nrmicro883 15100692

[5] Liu Y, Chen H, Kenney LJ, Yan J (2010) A divalent switch drives H-NS/DNA-binding conformations between stiffening and bridging modes. Genes & Development. 24(4):339–344. 10.1101/gad.1883510 20159954 PMC2816733

[6] van der Valk RA, Vreede J, Qin L, Moolenaar GF, Hofmann A, Goosen N, Dame RT (2017) Mechanism of environmentally driven conformational changes that modulate H-NS DNA-bridging activity. eLIfe. 6:e27369 10.7554/eLife.27369 28949292 PMC5647153

[7] Atlung T, Ingmer H. (1997) H-NS: a modulator of environmentally regulated gene expression. Molecular microbiology. 24(1):7–17. 10.1046/j.1365-2958.1997.3151679.x 9140961

[8] Rimsky S (2004) Structure of the histone-like protein H-NS and its role in regulation and genome superstructure. Current opinion in microbiology. 7(2):109–114. 10.1016/j.mib.2004.02.001 15063845

[9] Ono S, Goldberg M, Olsson T, Esposito D, Hinton J, Ladbury J (2005) H-NS is a part of a thermally controlled mechanism for bacterial gene regulation. Biochemical Journal. 15;391(2):203–13. 10.1042/BJ20050453 15966862 PMC1276917

[10] Navarre WW, Porwollik S, Wang Y, McClelland M, Rosen H, Libby SJ, Fang FC (2006) Selective silencing of foreign DNA with low GC content by the H-NS protein in Salmonella. Science. 313(5784):236–238. 10.1126/science.1128794 16763111

[11] Oshima T, Ishikawa S, Kurokawa K, Aiba H, Ogasawara N (2006) Escherichia coli histone-like protein H-NS preferentially binds to horizontally acquired DNA in association with RNA polymerase. DNA research. 13(4):141–153. 10.1093/dnares/dsl009 17046956

[12] Dorman CJ. (2007) H-NS, the genome sentinel. Nature Reviews Microbiology. 5(2):157–161. 10.1038/nrmicro1598 17191074

[13] Navarre WW, McClelland M, Libby SJ, Fang FC (2007) Silencing of xenogeneic DNA by H-NS?facilitation of lateral gene transfer in bacteria by a defense system that recognizes foreign DNA. Genes & Development. 21(12):1456–1471. 10.1101/gad.1543107 17575047

[14] Arold ST, Leonard PG, Parkinson GN, Ladbury JE (2010) H-NS forms a superhelical protein scaffold for DNA condensation. Proceedings of the National Academy of Sciences. 107(36):15728–15732. 10.1073/pnas.1006966107 PMC2936596 20798056

[15] Shindo H, Iwaki T, Ieda R, Kurumizaka H, Ueguchi C, Mizuno T, Morikawa S, Nakamura H, Kuboniwa H (1995) Solution structure of the DNA binding domain of a nucleoid-associated protein, H-NS, from Escherichia coli. FEBS letters. 360:125–131. 10.1016/0014-5793(95)00079-O 7875316

[16] Gordon BRG, Li Y, Cote A, Weirauch MT, Ding P, Hughes TR, Navarre WW, Xia B, Liu J (2011) Structural basis for recognition of AT-rich DNA by unrelated xenogeneic silencing proteins. Proc. Natl. Acad. Sci. USA. 108:10690–10695. 10.1073/pnas.1102544108 21673140 PMC3127928

[17] Dorman CJ, Hinton JC, Free A (1999) Domain organization and oligomerization among H-NS-like nucleoid-associated proteins in bacteria. Trends in microbiology. 1;7(3):124–8. 10.1016/S0966-842X(99)01455-9 10203842

[18] Smyth CP, Lundbäck T, Renzoni D, Siligardi G, Beavil R, Layton M, Sidebotham JM, Hinton JC, Driscoll PC, Higgins CF, Ladbury JE (2000) Oligomerization of the chromatin-structuring protein H-NS. Molecular microbiology. 36:962–972. 10.1046/j.1365-2958.2000.01917.x 10844682

[19] Gao Y, Foo YH, Winardhi RS, Tang Q, Yan J, Kenney LJ (2017) Charged residues in the H-NS linker drive DNA binding and gene silencing in single cells. Proc. Natl. Acad. Sci. USA. 114:12560 10.1073/pnas.1716721114 29109287 PMC5703333

[20] Cordeiro TN, Schmidt H, Madrid C, Juárez A, Bernadó P, Griesinger C, García J, Pons M (2011) Indirect DNA readout by an H-NS related protein: structure of the DNA complex of the C-terminal domain of Ler. 17;7(11):e1002380. 10.1371/journal.ppat.1002380 PMC3219716 22114557

[21] Ali SS, Xia B, Liu J, Navarre WW (2012) Silencing of foreign DNA in bacteria. Current opinion in microbiology. 15(2):175–181. 10.1016/j.mib.2011.12.014 22265250

[22] Yamada H, Yoshida T, Tanaka Ki, Sasakawa C, Mizuno T (1991) Molecular analysis of the Escherichia coli has gene encoding a DNA-binding protein, which preferentially recognizes curved DNA sequences. Molecular and General Genetics MGG. 230(1-2):332–336. 10.1007/BF00290685 1745240

[23] Owen-Hughes TA, Pavitt GD, Santos DS, Sidebotham JM, Hulton CS, Hinton JC, Higgins CF (1992) The chromatin-associated protein H-NS interacts with curved DNA to influence DNA topology and gene expression. Cell. 71(2):255–265. 10.1016/0092-8674(92)90354-F 1423593

[24] Lucchini S, Rowley G, Goldberg MD, Hurd D, Harrison M, Hinton JC (2006) H-NS mediates the silencing of laterally acquired genes in bacteria. 18;2(8):e81. 10.1371/journal.ppat.0020081 PMC1550270 16933988

[25] B Blot N, Bouffartigues E, Buckle M, Geertz M, Gualerzi CO, Mavathur R, Muskhelishvili G, Pon CL, Rimsky S, Stella S (2007) High-affinity DNA binding sites for H-NS provide a molecular basis for selective silencing within proteobacterial genomes. Nucleic acids research. 35(18):6330–6337. 17881364 10.1093/nar/gkm712 PMC2094087

[26] Bouffartigues E, Buckle M, Badaut C, Travers A, Rimsky S (2007) H-NS cooperative binding to high-affinity sites in a regulatory element results in transcriptional silencing. Nature structural & molecular biology. 14(5):441–448. 10.1038/nsmb1233 17435766

[27] Navarre WW (2010) H-NS as a defence system in Bacterial chromatin (pp. 251–322). Springer, Dordrecht.

[28] Ulissi U, Fabbretti A, Sette M, Giulidori AM, Spurio R (2014) Time-resolved assembly of a nucleoprotein complex between Shigella flexneri virF promoter and its transcriptional repressor H-NS. Nucleid Acid Research. 42(21):13039 10.1093/nar/gku1052 PMC4245942 25389261

[29] Japaridze A, Reveney S, Sobetzko P, Liubov S, Nasser W, Dietler G, Muskhelishvili G (2017) Spatial organization of DNA sequences directs the assembly of bacterial chromatin by a nucleoid associated protein Journal of Biological Chemistry. 292(18):7607 10.1074/jbc.M117.780239 28316324 PMC5418058

[30] de Vries R (2011) Influence of mobile DNA-protein-DNA bridges on DNA configurations: coarse-grained Monte-Carlo simulations. The Journal of chemical physics. 135(12):125104 10.1063/1.3636383 21974563

[31] Joyeux M, Vreede J (2013) A model of H-NS mediated compaction of bacterial DNA. Biophysical journal. 104(7):1615–1622. 10.1016/j.bpj.2013.02.043 23561538 PMC3617430

[32] Riccardi E, Wang JC, Liapis AI (2010) A molecular dynamics study on the transport of a charged biomolecule in a polymeric adsorbent medium and its adsorption onto a charged ligand. The Journal of chemical physics. 133(8):084904 10.1063/1.3473930 20815591

[33] Riccardi E, Wang JC, Liapis AI (2014) Modeling the construction of polymeric adsorbent media: Effects of counter-ions on ligand immobilization and pore structure The Journal of Chemical Physics. 140(8):084901 10.1063/1.4865910 24588192

[34] Morio J, Balesdent M (2014) A survey of rare event simulation methods for static input—output models. Simulation Modelling Practice and Theory. 1;49:287–304. 10.1016/j.simpat.2014.10.007

[35] Bolhuis PG, Chandler D, Dellago C, Geissler PL (2002) Transition path sampling: throwing ropes over rough mountain passes, in the dark. Annual review of physical chemistry 53(1):291–318. 10.1146/annurev.physchem.53.082301.113146 11972010

[36] van Erp TS (2007) Reaction Rate Calculation by Parallel Path Swapping. Physical Review Letters. 25;98(26):268301 10.1103/PhysRevLett.98.268301 17678132

[37] Lervik A, Riccardi E, van Erp TS (2017) PyRETIS: A well-done, medium-sized python library for rare events. Journal of Computational Chemistry. 38(28):2439–2451. 10.1002/jcc.24900 28749600

[38] Cabriolu R, Skjelbred KM, Bolhuis PG, van Erp TS (2017) Foundations and latest advances in replica exchange transition interface sampling. The Journal of Chemical Physics. 21;147(15):152722 10.1063/1.4989844 29055317

[39] Duan Y, Wu C, Chowdhury S, Lee MC, Xiong G, Zhang W, Yang R, Cieplak P, Luo R, Lee T, Caldwell J (2003) A point-charge force field for molecular mechanics simulations of proteins based on condensed-phase quantum mechanical calculations. Journal of computational chemistry. 24(16):1999–2012. 10.1002/jcc.10349 14531054

[40] Jorgensen WL, Chandrasekhar J, Madura JD, Impey RW, Klein ML (1983) Comparison of simple potential functions for simulating liquid water. The Journal of chemical physics. 79(2):926–935. 10.1063/1.445869

[41] Ricci CG, de Andrade AS, Mottin M, Netz PA (2010) Molecular dynamics of DNA: comparison of force fields and terminal nucleotide definitions. Journal of Physical Chemistry B. 114(30):9882–9893. 10.1021/jp1035663 20614923

[42] Cheatham TI, Miller J, Fox T, Darden T, Kollman P (1995) Molecular dynamics simulations on solvated biomolecular systems: the particle mesh Ewald method leads to stable trajectories of DNA, RNA, and proteins. Journal of the American Chemical Society. 117(14):4193–4194. 10.1021/ja00119a045

[43] Essmann U, Perera L, Berkowitz ML, Darden T, Lee H, Pedersen LG (1995) A smooth particle mesh Ewald method. The Journal of chemical physics. 103(19):8577–8593. 10.1063/1.470117

[44] Van Der Spoel D, Lindahl E, Hess B, Groenhof G, Mark AE, Berendsen HJ (2005) GROMACS: fast, flexible, and free. Journal of computational chemistry. 26(16):1701–1718. 10.1002/jcc.20291 16211538

[45] Hess B, Kutzner C, Van Der Spoel D, Lindahl E (2008) GROMACS 4: algorithms for highly efficient, load-balanced, and scalable molecular simulation. Journal of chemical theory and computation. 4(3):435–447. 10.1021/ct700301q 26620784

[46] Loken C, Gruner D, Groer L, Peltier R, Bunn N, Craig M, Henriques T, Dempsey J, Yu CH, Chen J, Dursi LJ (2010) SciNet: lessons learned from building a power-efficient top-20 system and data centre. In Journal of Physics: Conference Series (Vol. 256, No. 1, p. 012026). IOP Publishing.

[47] Hess B, Bekker H, Berendsen HJ, Fraaije JG (1997) LINCS: a linear constraint solver for molecular simulations. Journal of computational chemistry. 18(12):1463–1472. 10.1002/(SICI)1096-987X(199709)18:12<1463::AID-JCC4>3.0.CO;2-H

[48] Miyamoto S, Kollman PA (1992) SETTLE: an analytical version of the SHAKE and RATTLE algorithm for rigid water models. Journal of computational chemistry. 13(8):952–962. 10.1002/jcc.540130805

[49] Bussi G, Donadio D, Parrinello M (2007) Canonical sampling through velocity rescaling. The Journal of chemical physics. 126(1):014101 10.1063/1.2408420 17212484

[50] Parrinello M, Rahman A (1981) Polymorphic transitions in single crystals: A new molecular dynamics method. Journal of Applied physics. 52(12):7182–7190. 10.1063/1.328693

[51] Nosé S, Klein M (1983) Constant pressure molecular dynamics for molecular systems. Molecular Physics. 50(5):1055–1076. 10.1080/00268978300102851

[52] Humphrey W, Dalke A, Schulten K (1996) VMD: visual molecular dynamics. Journal of molecular graphics. 14(1):33–38. 10.1016/0263-7855(96)00018-5 8744570

[53] The PyMOL Molecular Graphics System, Version 1.8, Schrödinger, LLC. 2015.

[54] Riccardi E, Dahlen O, van Erp TS (2017) Fast Decorrelating Monte Carlo Moves for Efficient Path Sampling. Journal of Physical Chemistry Letters. 6;8(18):4456–60. 10.1021/acs.jpclett.7b01617 28857565

[55] Moqadam M, Lervik A, Riccardi E, Venkatraman V, and Alsberg BK, van Erp TS (2018) Local initiation conditions for water autoionization Proceedings of the National Academy of Sciences. 115(20):E4569–76. 10.1073/pnas.1714070115 PMC5960278 29712836

[56] von Hippel PH, Berg OG (1989) Facilitated target location in biological systems Journal of Biological Chemistry. 15;264(2):675–8. 2642903

[57] Hornak V, Abel R, Okur A, Strockbine B, Roitberg A, Simmerling C (2006) Comparison of multiple Amber force fields and development of improved protein backbone parameters Proteins: Structure, Function and Bioinformatics 65:712–725. 10.1002/prot.21123 PMC4805110 16981200

[58] Ivani I, Dans PD, Noy A, Pérez A, Faustino I, Hospital A, Walther J, Andrio P, Goñi R, Balaceanu A, Portella G, Battistini F, Gelpí JL, González C, Vendruscolo M, Laughton CA, Harris SA, Case DA, Orozco M (2016) Parmbsc1: a refined force field for DNA simulations Nature Methods 13:55–58. 10.1038/nmeth.3658 26569599 PMC4700514

[59] Amit R, Oppenheim AB, Stavans J (2003) Increased bending rigidity of single DNA molecules by H-NS, a temperature and osmolarity sensor. Biophysical journal. 84(4):2467–2473. 10.1016/S0006-3495(03)75051-6 12668454 PMC1302812

[60] Sette M, Spurio R, Trotta E. Brandizi C, Brandi A, Pon CL Barbato G Boelens R, Gualerzi CO (2009) Sequence specific recognition of DNA by the C-terminal domain of nucleoid-associated protein H-NS Journal of Biological Chemistry. 284:30453–30462. 10.1074/jbc.M109.044313 19740756 PMC2781600

[61] van der Valk RA, Vreede J, Crémazy F, Dame RT (2015) Genomic Looping: A Key Principle of Chromatin Organization. Journal of molecular microbiology and biotechnology. 24(5-6):344–359. 10.1159/000368851 25732337

[62] Rohs R, West SM, Sosinsky A, Liu P, Mann RS, Honig B (2009) The role of DNA shape in protein-DNA recognition. Nature. 461(7268):1248–1253. 10.1038/nature08473 19865164 PMC2793086

[63] Yamada H, Muramatsu S, Mizuno T (1990) An Escherichia coli protein that preferentially binds to sharply curved DNA. Journal of biochemistry. 108(3):420–425. 10.1093/oxfordjournals.jbchem.a123216 2126011

[64] Dame RT, Wyman C, Goosen N (2001) Structural basis for preferential binding of H-NS to curved DNA. Biochimie. 83(2):231–234. 10.1016/S0300-9084(00)01213-X 11278073

